# The XIAP inhibitor AZD5582 improves the treatment effect of microwave ablation on hepatocellular carcinoma

**DOI:** 10.3389/fimmu.2025.1482954

**Published:** 2025-01-23

**Authors:** Wenhui Wang, Fuyuan Wu, Zhe Wu, Mengfan Zhang, Qiang Lu

**Affiliations:** ^1^ Department of Ultrasound, West China Hospital of Sichuan University, Chengdu, China; ^2^ Tianfu Jincheng Laboratory, City of Future Medicine, Chengdu, China; ^3^ Department of Interventional Radiology, The First Affiliated Hospital of Zhengzhou University, Zhengzhou University, Zhengzhou, China

**Keywords:** XIAP, anti-tumor immunity, hepatocellular carcinoma, microwave ablation, residual tumor

## Abstract

**Background and purpose:**

Microwave ablation (MWA) is one of the first-line therapy recommended for early-stage hepatocellular carcinoma (HCC). However, the residual tumor, resulting from insufficient ablation, led to recurrence and metastasis of liver cancer. Novel combination strategies are urgently needed to enhance efficiency of MWA.

**Methods:**

We detected the expression of XIAP protein after ablation in primary liver cancer patients using immunohistochemistry. Then, we established *in vitro* and *in vivo* IMWA models to further detect XIAP expression. We established an *in vitro* IMWA model by heating HCC cell lines and, at the same time, applied the XIAP inhibitor AZD5582 and verified the proliferation, migration, and pro-apoptotic ability of the XIAP inhibitor on tumor cells using CCK8, colony formation assay, cell scratch assay, and flow cytometry flow. The IMWA model of C57BL/6 and NTG mice were established, and AZD5582 was used in combination to evaluate the inhibitory and pro-apoptotic effects of different treatment regimens on tumor growth and to detect the local immune infiltration of C57BL/6 tumors. Finally, AZD5582 drug toxicity was detected to confirm its feasibility.

**Results:**

XIAP protein expression is significantly increased in recurrent hepatocellular carcinoma tissues of patients who previously received microwave ablation therapy. *In vitro* experiments showed that the migration and proliferation ability of HCC cells was significantly reduced, and the level of apoptosis was increased after application of the XIAP inhibitor AZD5582. *In vivo* experiments further confirmed that ablation combined with the application of AZD5582 significantly reduced the proliferation ability of residual hepatocellular carcinoma. Concurrently, in C57 BL/6 mice with AZD5582 application, the level of local CD8+ T-cell infiltration in the tumor was increased, while the level of Foxp3+ regulatory T-cell infiltration was significantly reduced. The low toxicity of AZD5582 was further confirmed through hematological and pathological examinations of vital organs. These results provide new clues for hepatocellular carcinoma treatment, suggesting the potential role of XIAP inhibitors in hepatocellular carcinoma treatment and their impact in immunomodulation.

**Conclusions:**

In this study, we found that the XIAP inhibitor AZD5582 modulates the immune microenvironment and inhibits the progression of post-ablation residual hepatocellular carcinoma.

## 1 Introduction

Hepatocellular carcinoma (HCC) is one of the most common malignant tumors worldwide and one of the leading causes of cancer-related deaths (1). Microwave ablation (MWA) has been shown to be an effective local therapeutic option with curative effects in patients with HCCs smaller than 3 cm in diameter (2). However, due to the uneven spatial distribution of heat energy across tumor tissue during MWA, the distal or terminal cancer cells survive and leads to local recurrences (3, 4). The recurrent cancer exhibits more aggressive phenotypes and unfavorable prognosis (5–7). Further research and innovation, especially in systemic medication, are needed to improve the therapeutic efficacy and survival rate for patients with HCC.

Thermal ablation damage is divided into three zones as follows: the central high-temperature zone, the sub-lethal temperature transition zone, and the surrounding normal tissue. In the transition zone, tumors suffer reversible damage and eventually survives leading to rapid tumor progression to an activated state (8). The anti-apoptotic proteins Bcl-XL, Mcl-1, c-IAP1, XIAP, and survivin are highly expressed in HCC, especially at the ablation margins (9). In addition, the ablated tumor margins also lead to several signaling pathways, activation of the PI3K/Akt signaling pathway, generation of PIP3, which in turn activates Akt and exerts an anti-apoptotic effect by phosphorylating a variety of downstream target proteins (e.g., Bcl-2-associated death-promoting proteins, mammalian target proteins of rapamycin) (10). Activation of the NF-κB signaling pathway promotes anti-apoptosis by regulating the generation of the IAP family of apoptosis inhibitory proteins, a family of Bcl-2 family proteins, from multiple downstream targets (11). The hypoxic conditions caused by ablation activate HIF-1α, which induces the expression of VEGF, EPO, Glut-1, and BNIP3, and exerts an anti-apoptotic pathway by regulating angiogenesis and energy metabolism (12). Therefore, the application of systemic treatment has been proposed to improve the therapeutic effect and survival rate for patients at high risk of recurrence (13). Currently, the expression profile of XIAP in the progression of residual cancer tissue after IMWA has not been reported, and the effect of XIAP in the progression of residual cancer tissue is not clear. The effect of XIAP inhibition in combination with thermal ablation on hepatocellular carcinoma is elusive. Therefore, the aim of this study was to evaluate the effect of pharmacological inhibition of XIAP in combination with MMA on hepatocellular carcinoma treatment *in vitro* and *in vivo*.

In this study, we evaluated the protein expression of XIAP in HCC tissues of patients. The *in vivo* effect of the XIAP inhibitor AZD5582 on cell proliferation, migration, and apoptosis of HCC cell lines was investigated. Furthermore, the *in vitro* effect of AZD5582 in combination with MWA was investigated on HCC xenografts of mice models.

## 2 Materials and methods

### 2.1 Human samples

Human HCC paraffin sections were from West China Hospital of Sichuan University (Chengdu, China), mainly including primary liver cancer that recurred after MWA and primary liver cancer that did not receive MWA. All patients provided written informed consent.

### 2.2 Cell culture and reagents

HCC cell lines (Hepa1-6 and Huh7) were purchased from Servicebio Technology Co., Ltd., China. Cells were cultured in Dulbecco’s modified Eagle’s medium (DMEM, Gibco, USA) supplemented with 10% fetal bovine serum (FBS: Excell, USA) and antibiotics (Gibco, USA): 100 U/ml of penicillin and 10 µg/ml of streptomycin in a humidified atmosphere of 5% CO_2_ at 37°C. After reviewing the literature and conducting pre-tests, we finally determined that the lowest effective concentration of AZD5582 (MCE, USA) in HCC cell lines was 25 μM.

### 2.3 *In vitro* imitated incomplete ablation and AZD5582 intervention

Insufficient MWA was simulated *in vitro* as follows: the HCC cell lines (Hepa1-6 and Huh7) were seeded in flasks and incubated for 24 h, after which the flasks were submerged in a 47°C water bath for 3 min. The cell lines were divided into four groups as follows: the control group (cells were not subjected to any treatment), Heat group (47°C water bath for 3 min), AZD5582 group (cells were treated with AZD5582 at a concentration of 25 µm/L), and Heat + AZD5582 group (cells were treated with AZD5582 medium at a concentration of 25 µm/L after heating in a water bath at 47°C for 3 min).

### 2.4 Cell proliferation assay

Cells were seeded in 96-well plates (1 × 10^4^/well) and allowed to settle down for 24 h in accordance with the above grouping. After 24 h, 10 μl of the CCK-8 reagent (MCE, USA) was added to each well. After incubation for 1 h at 37°C, absorbance measurements were performed at 450 nm using a micro-plate reader (Bio Tek, USA). The proliferation of each group of cells was assessed by measuring the absorbance value.

### 2.5 Colony formation assay

For colony formation assay, 1.5 × 10^3^ cells per well were seeded into six-well plates for 24 h in accordance with the above grouping. After incubation for 72 h, each well was refreshed with new full medium every 3 days. After being cultured for 7 days, the cells were fixed with 4% paraformaldehyde and then stained with crystal violet dye (Servicebio, China). Afterward, the plates were washed with ddH2O several times, dried, and photographed.

### 2.6 Cell scratch assay

A total of 2 × 10^5^ cells of each cell line were seeded out in six-well plates. When cells grew to reach a tight cell monolayer in a six-well plate, cells were treated according to the above grouping. The cell monolayer was scratched with a plastic pipette tip. The remaining cells were washed twice with PBS. After 48 h, the migrated cells at the wound front were photographed using an Olympus microscope and analyzed using ImageJ software.

### 2.7 Apoptosis analysis (flow cytometry)

Cells were treated according to the above grouping. The apoptosis is performed following the manufacturer’s protocol of a double staining kit of Annexin V-FITC and PI (Yeasen, China). Cells were trypsinized (Gibco,USA) and washed twice with 1× PBS. The cell density was adjusted to 1 × 10^6^ cells per ml. Cells were resuspended with 500 μl of 1× Binding Buffer containing 5 μl of Annexin V-FITC and 10 μl of PI, and then incubated for 15 min in the dark. The percentage of apoptotic cells was detected using a Flow Cytometer (Beckman Coulter, USA).

### 2.8 Quantitative real-time PCR analysis

HCC cell lines (Hepa-6, Huh7) were each divided into two groups, control and heated. Incubation was continued in the incubator for 24 h after treatment, and RNA was extracted from the cells. Gene expression levels were quantified using real-time reverse transcription polymerase chain reaction (RT-qPCR). Total mRNA was isolated from cells using Tri-reagent (Invitrogen, California) according to the manufacturer’s protocol. The mRNA was then used to synthesize cDNA using the cDNA synthesis kit (Vazyme, China). Quantitative PCR was performed according to a standard protocol using the SYBR Green Real-Time PCR MasterMix (novoproten, China) in a QuantStudio™ 6. Relative mRNA expression levels of the gene of interest were calculated using the 2−△△Ct method. The housekeeping RPS18 served as an internal control to normalize the mRNA expression levels of the target genes. The sequences of the primers used for PCR analysis are shown in (**Table 1**).

**Table 1:** Primers for RT-qPCR.

Species	Gene	Forward primer	Reverse primer
Mouse	Rps18	TGGGAAGTACAGCCAGGTTC	AGTGGTCTTGGTGTGCTGAC
	Xiap	GAGGGCTCACGGATTGGAAG	TCCAATAGGTATTTGCACCCTG
	Cd4	TCCTAGCTGTCACTCAAGGGA	TCAGAGAACTTCCAGGTGAAGA
	Cd8	CAAGCCCAGACCTTCAGAGA	TCCCCATCACACCCCTACTA
	Foxp3	CTCGTCTGAAGGCAGAGTCA	TGGCAGAGAGGTATTGAGGG
Human	RPS18	TGCGAGTACTCAACACCAACA	CTTCGGCCCACACCCTTAAT
	XIAP	TGCAAGAGCTGGATTTTATGC	GGTCTTCACTTGGCTTCCAAT

## 3 *In vivo* experiments

### 3.1 Animal care and tumor model establishment

Male C57BL/6J mice and male NTG mice (5 to 6 weeks old, 18–26 g) were provided by GemPharmatech (Nanjing, China). All mice were housed in a specific pathogen-free facility accredited by the Association for Assessment and Accreditation of Laboratory Animal Care International. All animal welfare and experimental procedures were approved by the Ethics Committee of Laboratory Animals, West China Hospital, Sichuan University. The mice were maintained on a 12-/12-h light/dark cycle at 21°C–25°C and 40%–70% humidity with access to sterile pellet food and water *ad libitum*. Animals were labeled by ear numbers throughout the experimental period, and each animal had a unique animal number. Hepa1-6 cells (1 × 10^6^) or Huh7 cells (1 × 10^6^) were injected subcutaneously into the right thighs of male C57 BL/6 mice or male NTG, respectively. When tumor diameter reached 0.7–0.8 cm, a total of 20 mice were randomly assigned into four groups: the control, IMWA, AZD5582, and combination of IMWA and AZD5582 groups. The length diameter and width of the tumor were measured daily. Tumor volumes were determined using the following equation: volume (mm^3^) = length × width × width × 0.5.

### 3.2 Tumor treatment

We divided 10 male C57BL/6J mice with loaded tumors into control and IMWA groups of five mice each. Mice in the control group were not subjected to any treatment, while mice in the IMWA group were subjected to microwave ablation. The specific method was as follows: after anesthesia, mice in the IMWA group were anesthetized, a small incision was made in the tumor, and a microwave ablation needle was used to penetrate the incision into the tumor tissue, and the ablation was performed at a power of 5 W for 30 s. After 4 days of treatment, the tumors were removed for immunofluorescence detection to observe XIAP protein expression.

To prepare the AZD5582 working solution for the *in vivo* experiments, the appropriate amount of AZD5582 powder was first weighed, and then each of the following reagents was added sequentially: 10% DMSO, 40% PEG300, 5% Tween-80, and 45% saline and mixed well.

Mice in the AZD5582 group were administered with 0.3 mg/kg of AZD5582 solution by intraperitoneal injection once in 2 days for 12 days, while the control group was administered with the same amount of saline for 12 days. Mice in the IMWA group were treated as described above, and feeding was continued for 12 days. Mice in the combination group were treated by IMWA as stated above and administered with 0.3 mg/kg of AZD5582 solution by intraperitoneal injection once in 2 days for 12 days. At the end of the experiment, tumor tissues were harvested, imaged, and their volumes were determined. Samples were stored at −80°C until subsequent studies. Vital organs (liver, heart, lungs, kidneys, and spleen) of mice were collected and fixed in 4% phosphate-buffered paraformaldehyde for subsequent assessment of post-treatment organ toxicity. Body weights were measured at the indicated ages during the entire experimental period.

### 3.3 Histopathological analysis

Tumor tissues were fixed with 4% paraformaldehyde and then paraffin embedded, and sections with a thickness of 4 µm were cut from the largest cross-section of the tumor and stained with hematoxylin–eosin (H&E). Finally, the tumor necrosis rate of NTG mice was calculated as = (area of tumor necrosis in the largest cross-section/area of the whole tumor) × 100% to calculate the NTG tumor necrosis rate. Immunohistochemical staining was performed using the DAB staining method, and the primary antibodies applied included anti-XIAP (dilution 1:200, Cell Signaling) antibodies. The nuclei of cells in tissue sections were restained blue with hematoxylin. The TUNEL kit (Thermo Fisher) was performed to identify apoptosis in treated/untreated HCC cells. After the prescribed treatment according to the manufacturer’s protocol, DAPI was utilized to stain the nucleus (blue), and TUNEL staining (green) was applied to label apoptotic cells. Finally, fluorescence microscopy (Nikon) was employed to determine the number of TUNEL-positive cells after magnified imaging.

The multiplex immunofluorescence staining method allows for the analysis of the differential expression of protein markers with multiple markers in the same section (14). After deparaffinization with xylene and hydration with a gradual series of ethanol, antigen retrieval was performed with boiling in antigen retrieval solution EDTA. Endogenous peroxidase was inactivated by incubation in 3% H_2_O_2_ for 15 min. Next, the sections were pre-incubated with 10% normal goat serum and then incubated for 2 h or overnight with the following primary antibodies: CD4 (1:200 dilution, Servicebio), CD8 (1:200 dilution, Servicebio), Foxp3 (1:200 dilution, Servicebio), followed by the addition of horseradish peroxidase (HRP)-conjugated secondary antibody (Ab) at room temperature for 30 min. The antigenic binding sites were visualized using OPAL dye. Tyramine signal amplification (TSA) visualization was then performed with the opal color multiplex immunohistochemistry kit (PerkinElmer, USA) containing fluorophores (DAPI), Opal 520 (CD4), Opal 570 (CD8), and Opal 620 (Foxp3). Slides were scanned using the PerkinElmer Vectra (PerkinElmer, US).

### 3.4 Quantitative real-time PCR analysis

The experimental method is the same as the RT-qPCR experimental procedure for *in vitro* experiments.

### 3.5 Blood analysis

Peripheral blood samples were collected from the retro-orbital sinus after the experiment. Red blood cell (RBC), white blood cell (WBC), platelet (PLT), glutamic pyruvic transaminase (ALT), glutamic oxaloacetic transaminase (AST), and creatinine (CREA) were detected using a hematology analyzer (Automatic Blood Cell Analyzer, Mindray) and an animal biochemical analyzer (Automatic Biochemistry Analyzer, Mindray).

### 3.6 Statistical analysis

Data were expressed as mean ± standard deviation (mean ± SD) or mean ± standard error of the mean (mean ± SEM). Comparisons between multiple groups were made using analysis of variance (ANOVA). Additional *post hoc* analyses were performed using unpaired Student’s t-test when ANOVA reaches statistical differences. Comparisons between the two groups were analyzed using the Mann–Whitney test for non-normal distribution. A value of p < 0.05 was considered statistically different. Data analysis was conducted using GraphPad Prism 8 (*p < 0.05; **p < 0.01; ***p < 0.001; ns: p > 0.05).

## 4 Results

### 4.1 XIAP is increased in the residual cancer tissue after ablation

**Figure 1:**
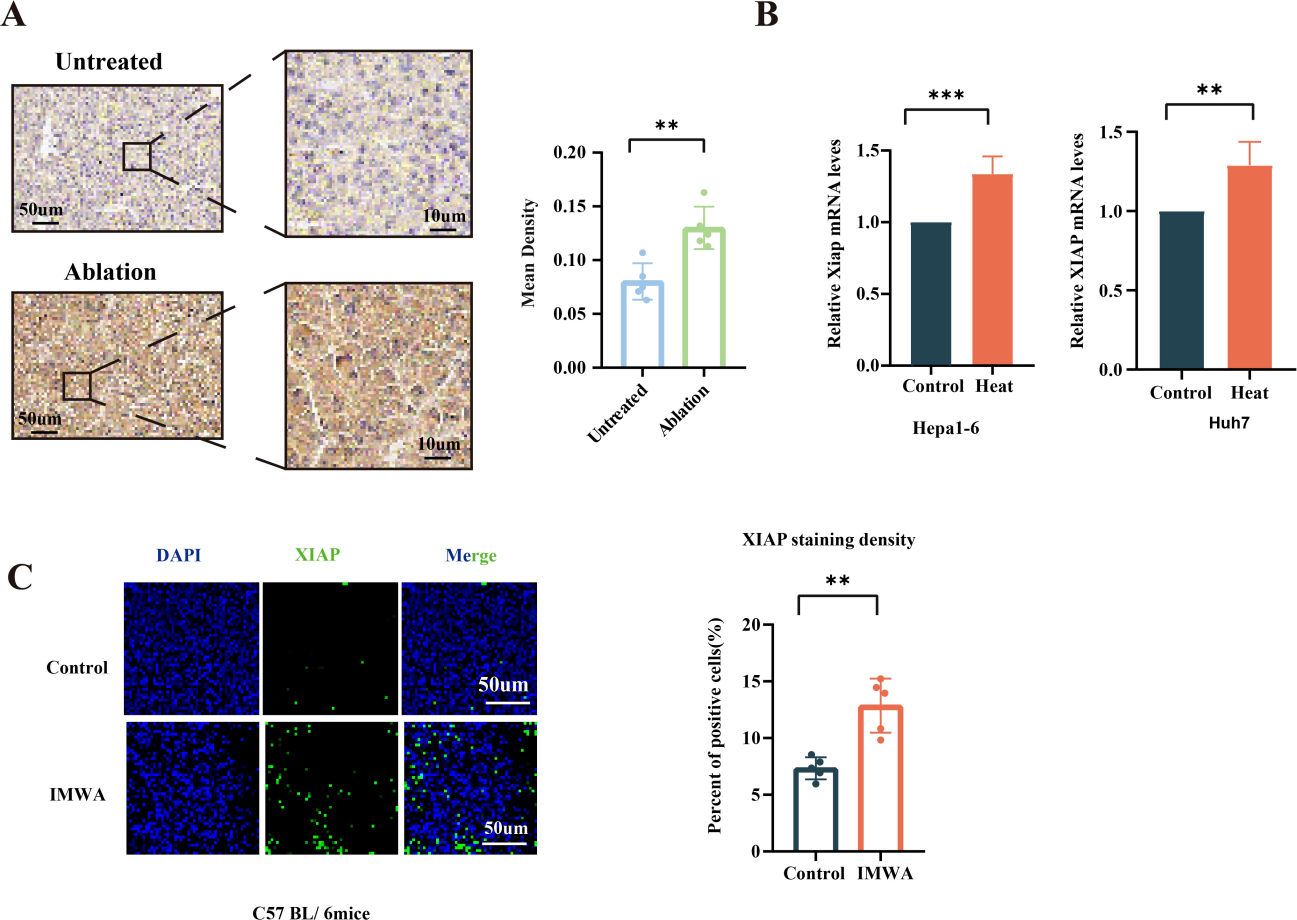
Expression of XIAP was increased after thermal ablation both *in vivo* and *in vitro*. **(A)** IHC results of tissue slices from patients who underwent microwave ablation (n = 5) compared to untreated patients (n = 5). **(B)** The mRNA expression of XIAP in heat-treated HCC cells. **(C)** Immunofluorescence of XIAP protein in the xenografts of mice models at 4 days post-surgery (**p < 0.01, ***p < 0.001).

XIAP protein expression was measured on the paraffin-embedded sections via IHC (**Figure 1A**). The quantified results of images indicated that the protein expression of XIAP was increased in the recurrent liver cancer tissue of patients who previously received MWA treatment. In addition, mRNA expression of XIAP was measured in the *in vitro* heat-treated Hepa1-6 cells and Huh-7 cells. Results revealed that the mRNA expression of XIAP was increased in both cells (**Figure 1B**). The Hepa1-6 cell-derived subcutaneous xenograft of C57/BL6 mice were treated with insufficient MWA to evaluate the XIAP expression in the residual cancer tissue. The results indicated that XIAP protein was increased in the residual cancer tissue (**Figure 1C**). Collectively, the results demonstrated that XIAP was increasingly expressed in the recurrent human HCC tissue and experimental HCC models treated with insufficient MMA.

### 4.2 AZD5582 enhances the antitumor effect of IMWA *in vitro*

**Figure 2:**
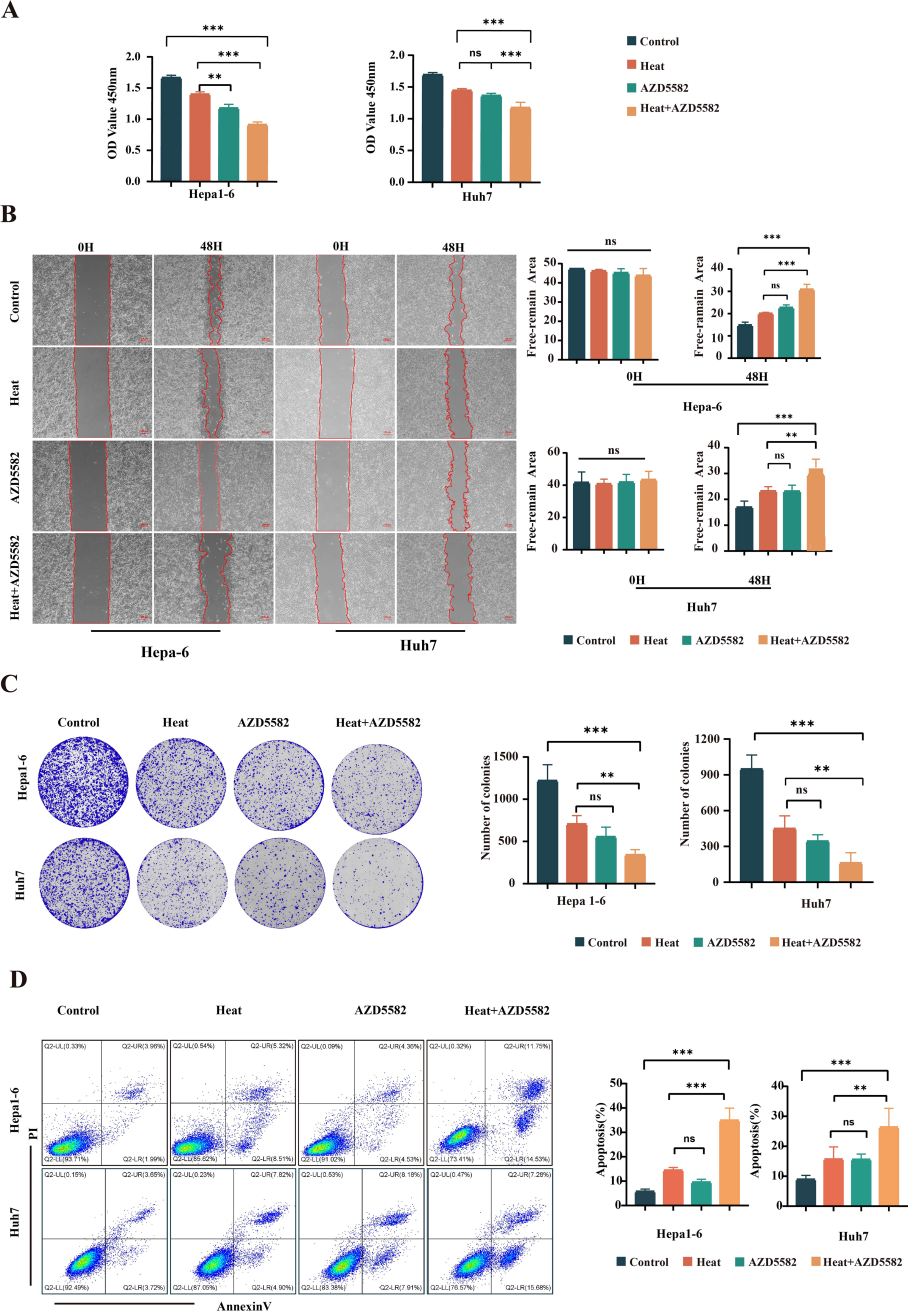
AZD5582 exhibits significant antitumor effects in an *in vitro* HCC cell line ablation model. **(A)** CCK8 assay detects the activity of cells in each treatment group. **(B)** Cell scratch assay detects the invasive ability of cells in each group after treatment. **(C)** Colony formation assay shows the cell proliferation ability between the treatment groups. **(D)** Apoptosis assay shows the ability of cells in each group to promote apoptosis after undergoing different treatment steps (ns: p > 0.05, **p < 0.01, ***p < 0.001).

The XIAP inhibitor AZD5582 was applied in the *in vitro* model of IMWA. The cell viability assay showed that AZD5582 synergistically with heat treatment decreased the cell viability of Hepa1-6 cells and Huh7 cells (**Figure 2A**). Cell scratch assay showed that the combination of AZD5582 and heat treatment inhibited cell migration to the most extent (p < 0.001) (**Figure 2B**). The effect of XIAP inhibition on cell proliferation was evaluated with colony formation assay. The results demonstrated that the combination of AZD5582 and heat treatment showed the most significant inhibitory effect on the proliferation of Hepa1-6 cells and Huh7 cells (**Figure 2C**). Furthermore, AZD5582 in combination with heat treatment showed the strongest apoptosis-inducing effect on Hepa1-6 cells and Huh7 cells, which was revealed by Annexin V/PI double staining flow cytometry (**Figure 2D**). These results showed that AZD5582 synergistically combined with heat treatment significantly induced apoptosis of liver cancer cells and inhibited the proliferation and migration of HCC cells.

### 4.3 IMWA combined with AZD5582 reduces tumor volume of subcutaneous HCC cells in mice

**Figure 3:**
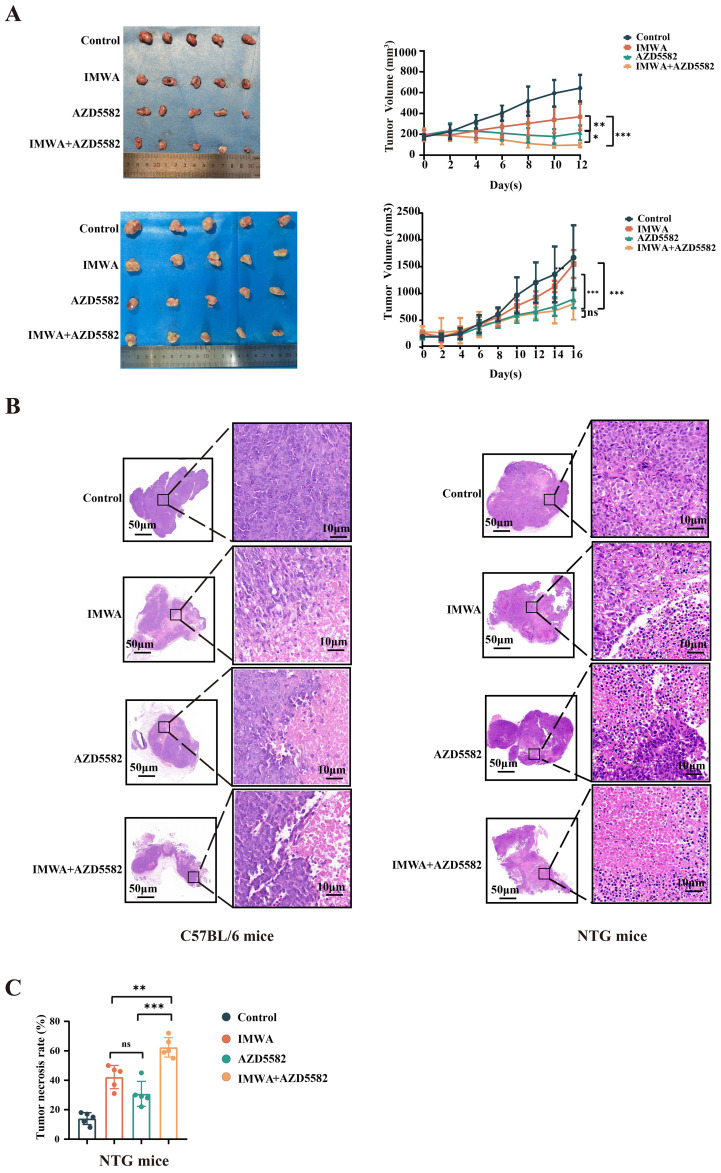
The combination of AZD5582 with incomplete microwave ablation exhibits anti-tumor effects in a mouse model of HCC. **(A)** The representative tumors, which were harvested from different groups, presented with the smallest tumor size in the combined group. **(B)** Tumor tissue with hematoxylin and eosin staining. **(C)** Tumor necrosis rate in NTG mice in each treatment group after treatment (ns: p > 0.05, *p < 0.05, **p < 0.01, ***p < 0.001).

C57BL/6 and NTG mice were subjected to collection of subcutaneous tumors 12–16 days after treatment according to the experimental design. At the end of the treatment, the mean tumor volume of C57BL/6 mice of the IMWA group was slightly larger than that of the AZD5582 group (p < 0.01). The AZD5582 in combination with IMWA group had the remarkably smallest mean tumor volume, which was even slightly smaller than before the AZD5582 treatment (**Figure 3A**). In NTG mice, the mean tumor volume of each group was gradually increased after the treatment. At the final measurement, the mean tumor volume of the IMWA group was close to that of the control group. However, AZD5582 treatment, with or without IMWA, reduced the mean tumor volume compared to the control group (**Figure 3B**). An overview of HE staining of the liver cancer tissue revealed that none of the treatment strategies induced obvious necrosis of the cancer tissue (**Figure 3B**). Tumor necrosis of NTG mice was measured via HE staining, and the mean necrosis rate of the IMWA + AZD5582 group was significantly higher than in the other three groups (**Figure 3C**). Altogether, the results demonstrated that the XIAP inhibitor AZD5582 in combination with IMWA drastically inhibited the progression of liver cancer tissue in immunity-intact mice, and the XIAP inhibitor also inhibited the progression of liver cancer tissue in immunity-deficient mice.

### 4.4 IMWA combined with AZD5582 inhibits cell proliferation and promotes cell apoptosis of cancer tissue in mice

**Figure 4:**
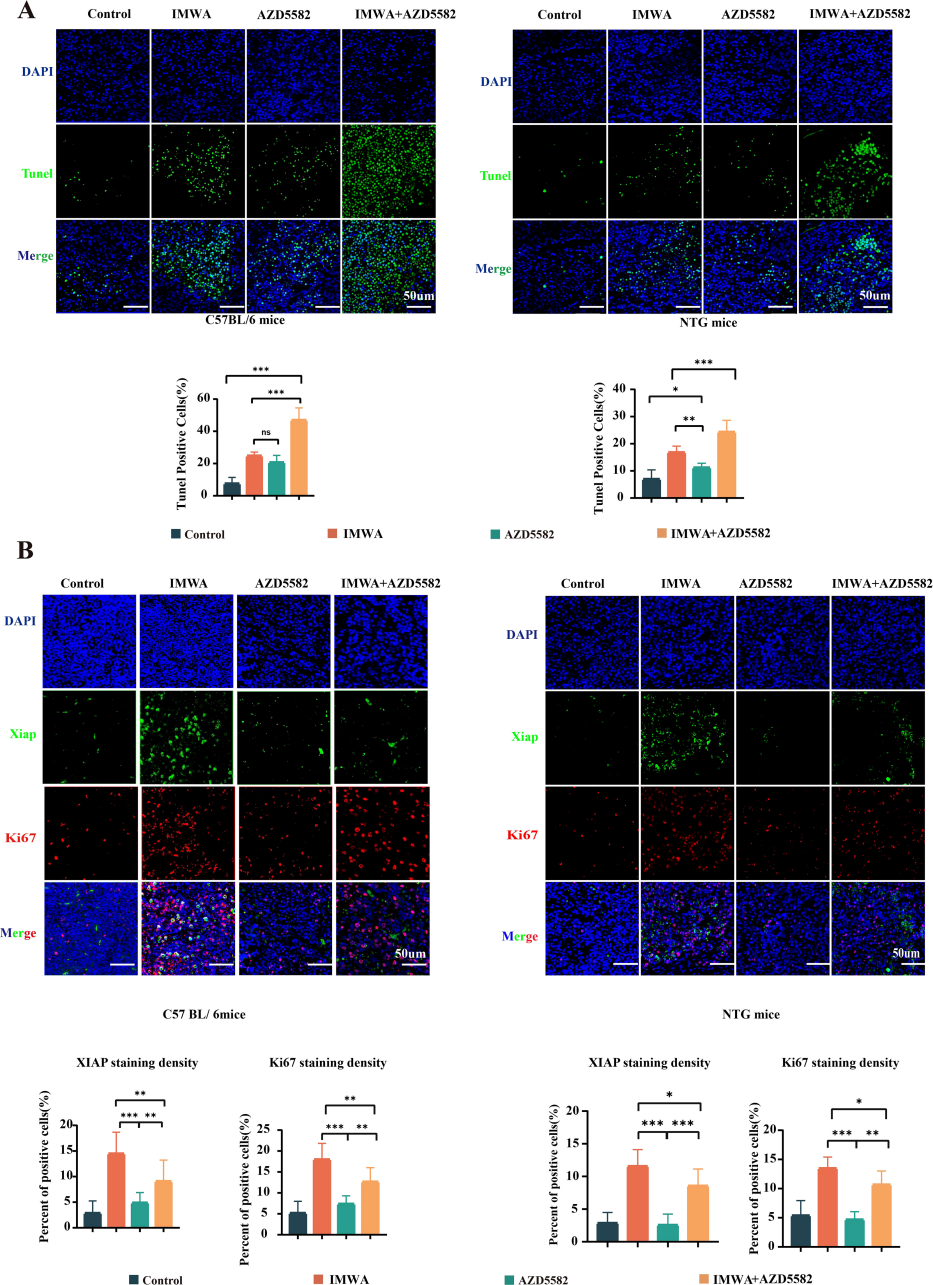
Pathologic analysis of the tumors in four treatment groups. **(A)** TUNEL staining on the cancer tissue of C57BL/6 mice and NTG mice. **(B)** Double staining of Ki-67 and XIAP on the cancer tissue of C57BL/6 mice and NTG mice (scale bars, 50 µm) (*p < 0.05, **p < 0.01, ***p < 0.001).

To figure out the mechanism of XIAP inhibitor suppressing the progression of cancer tissues in mice models, TUNEL staining was performed to evaluate cell apoptosis of the cancer tissues (**Figure 4A**). Quantified results showed that compared to the control group, either IMWA or AZD5582 increased the apoptotic cell ratio of cancer tissue in both C57BL/6 mice and NTG mice. Notably, the combination of IMWA and AZD5582 induced the largest percentage of apoptotic cells, which was more than 40% and more than 20%, respectively, in the cancer tissues of C57BL/6 mice and NTG mice. Ki-67 is one of the most widely used cell proliferation marker in clinical pathology to predict the progression of cancer tissue. Double staining of Ki-67 and XIAP was performed to evaluate the treatment effectiveness of IMWA and AZD5582 on cancer tissues (**Figure 4B**). The expression of both Ki-67 and XIAP was significantly increased in the IMWA-treated cancer tissues of C57BL/6 mice and NTG mice. The combination of AZD5582 and IMWA decreased the expression of Ki-67 and XIAP compared to IMWA alone. The results demonstrated the preferable treatment effect of the combination of IMWA and AZD5582 to monotherapy via inducing cell apoptosis and cell proliferation of cancer tissues in C57BL/6 mice and NTG mice. In addition, the application of AZD5582 mitigated the aggressive phenotype of post-ablation cancer tissue induced by IMWA in the mice models.

### 4.5 AZD5582 modulates the immune microenvironment of residual tumors after IMWA treatment

**Figure 5:**
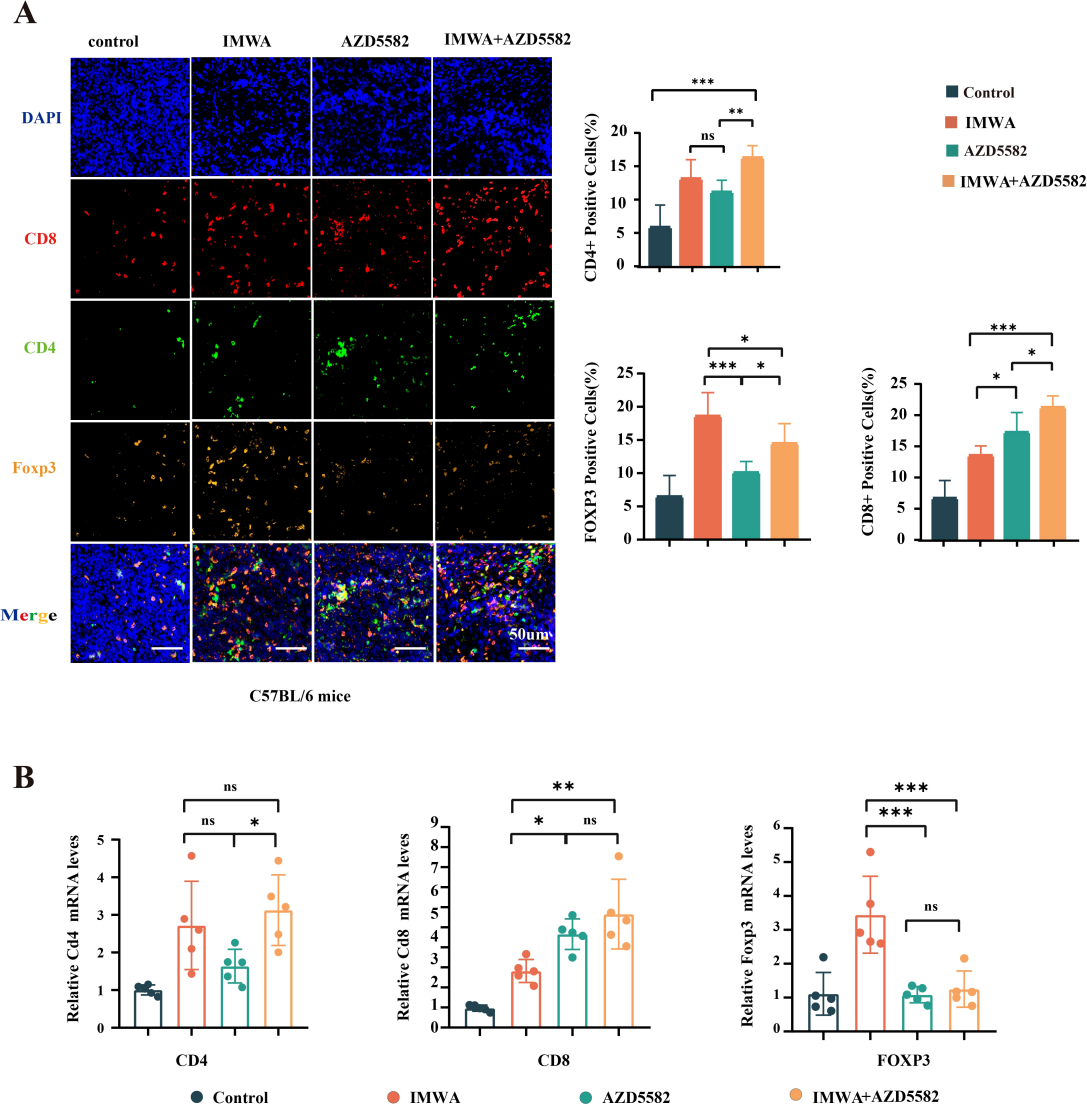
Analysis of the local immune microenvironment of tumors in four treatment groups. **(A)** The multiplex immunofluorescence staining of CD8, CD4, and Foxp3 on the cancer tissues of C57BL/6 mice. **(B)** mRNA expression of CD4, CD8, and Foxp3 in the cancer tissues of C57BL/6 mice (ns: p > 0.05, *p < 0.05, **p < 0.01, ***p < 0.001).

In the previous results, the combination of AZD5582 and IMWA showed a surprisingly inhibitory effect on the tumor volume in the immunity-intact C57BL/6 mice compared to the immunity-deficient NTG mice. AZD5582 was reported to improve the anti-tumoral effect via enhancing the killing function of immune cells. Therefore, we assumed that AZD5582 could regulate T-cell function in the cancer tissue of C57BL/6. The common markers of effector T cells, helper T cells, and regulatory T cells, which were CD8, CD4, and Foxp3, respectively, were stained on the cancer tissue sections of C57BL/6 mice (**Figure 5A**). The results revealed that the combination of AZD5582 and IMWA recruited the most abundant CD8-positive T cells, which was up to approximately 20% in the TME of cancer tissues. The ratio of Foxp3-positive immune-suppressive regulatory T cells was increased in the TME of cancer tissues treated with IMWA alone but was decreased by the additional application of AZD5582. In addition, the mRNA expression of CD8, CD4, and Foxp3 was measured with RT-qPCR to evaluate the overall T-cell infiltration in the TME of cancer tissues (**Figure 5B**). The mRNA expression of CD8 in cancer tissues treated with the combination of AZD5582 and IMWA was approximately fourfold higher than that of the control group. The mRNA expression of Foxp3 was increased by twofold in the cancer tissues treated with IMWA alone but completely reverted to their normal state with the additional application of AZD5582. The results showed that the XIAP inhibitor AZD5582 regulated the tumor immune microenvironment of cancer tissues in C57BL/6 mice.

### 4.6 Evaluation of *in vivo* toxicity of AZD5582

**Figure 6:**
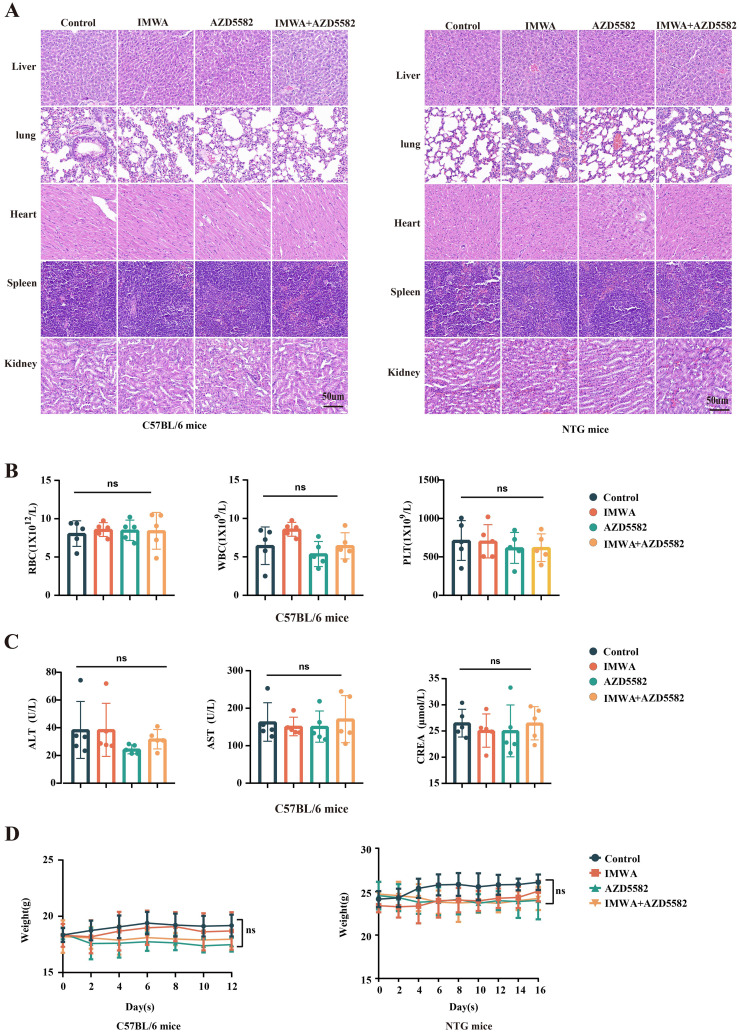
The safety assessment of treatment for residual tumors after IMWA of HCC. **(A)** Histopathological observations of vital organs in mice under different treatment regimens. **(B)** Analysis of blood cell counts in C57BL/6 mice under different treatment regimens. **(C)** Analysis of liver and kidney functions in C57BL/6 mice under different treatment regimens. **(D)** The bodyweight of C57BL/6 mice and NTG mice during the treatment (ns: p > 0.05).

To evaluate the toxicity of *in vivo* use of the XIAP inhibitor AZD5582, the HE staining of multiple organs, peripheral blood cells count, and serum markers of liver function and kidney function were investigated. No obvious toxicity-related pathological changes, evaluated with HE staining, were observed in the livers, hearts, spleens, lungs, and kidneys of both C57BL/6 mice and NTG mice in our study (**Figure 6A**). The counts of red blood cells (RBC), white blood cells (WBC), and platelets were not affected by AZD5582 in the C57BL/6 mice, indicating that AZD5582 did not induce obvious hematopoietic toxicity (**Figure 6B**). In addition, the serum concentration of liver injury markers, including ALT and AST, and kidney injury marker CREA, was not influenced by AZD5582. Furthermore, no obvious reduction in bodyweight of both C57BL/6 mice and NTG mice was obvious in the experiment (**Figure 6C**). In conclusion, the application of XIAP inhibitor AZD5582 showed preferable safety in the study (**Figure 6D**).

## 5 Discussion

In this study, we demonstrated that the expression of XIAP protein is increased in recurrent HCC tissues of patients who previously received MWA treatment. This indicates that XIAP plays a significant role in the progression of liver cancer after MWA treatment.

X-linked inhibitor of apoptosis protein (XIAP), also known as inhibitor of apoptosis protein 3 (IAP3), and human IAPs, like protein (hILP), is an apoptosis-inhibitory protein involved in the carcinogenesis, progression, and metastasis of tumors (15, 16). Human XIAP protein is encoded by the XIAP gene located at Xq24-25 and ubiquitously expressed in various tissues, such as the heart, brain, and lungs. XIAP is a protein with a molecular weight of 57 kDa containing three BIR domains, a ubiquitin-binding domain (UBA), and a RING finger domain (17). XIAP inhibits apoptosis by regulating the activity of cysteine asparaginase. XIAP selectively binds to, and inhibits, caspases-3, caspases-7, and caspases-9 to prevent apoptosis from proceeding normally, and antagonizes the activity of these two enzymes using the interaction of the BIR1 and BIR2 regions with activated caspase-3 and caspase-7 (13, 18). XIAP utilizes the interaction of the BIR3 region with caspase-9 to prevent caspase-9 from generating an active dimeric structure. In addition to anti-apoptotic function, XIAP is also found involved in autophagy, necroptosis, and copper homeostasis maintenance (18). It has been shown that a high expression of XIAP is associated with poor prognosis and drug resistance in a variety of tumor types (18, 19).

The XIAP inhibitor AZD5582 and heat treatment synergistically induce apoptosis of liver cancer cells, and inhibit proliferation and migration of liver cancer cells *in vitro*. The *in vivo* use of AZD5582 improves the treatment effect of IMWA on liver cancer cell line-derived xenograft mice models via inducing cell apoptosis and inhibiting cell proliferation without obvious systemic toxicity. In addition, AZD5582 enhances the infiltration of CD8-positive T cells and decreases the infiltration of Foxp3-positive Treg cells in the TME of cancer tissues of immune-intact C57BL/6 mice. This shift in immune cell composition may further contribute to the anti-tumor effects observed with AZD5582 treatment.

AZD5582 is a small molecule inhibitor targeting XIAP with anti-tumor potential. A few studies indicate that inhibition of XIAP can be used for anti-cancer therapy for liver cancer (20, 21). We find a significant increased expression of XIAP in the recurrent liver cancer. Likewise, XIAP is also increased in the residual cancer tissues of preclinical mice models that received IMWA treatment. Therefore, we assume that a pharmacological XIAP inhibitor may improve the anti-tumoral effect of IMWA.

There are several main classes of inhibitors targeting XIAP as follows: 1) SMAC/DIABLO mimics (LCL161 and AZD5582), which mimic natural SMAC/DIABLO proteins by binding to the BIR structural domain of XIAP. They competitively block the interaction of XIAP with caspases and lift their inhibition (22). 2) Low-molecular weight compounds (Embelin), which promote apoptosis by binding to the BIR3 structural domain of XIAP and preventing its inhibition of caspase-9, thereby inhibiting its function (23). Current studies on XIAP inhibitors for HCC have shown that LCL161 demonstrated synergistic effects with paclitaxel on HCC cells by modulating BCL-2, while Embelin modulated apoptosis and cell cycle by inhibiting XIAP and thus regulating cyclin D1 (22, 23). AZD5582 is a dual IAP inhibitor, which not only inhibits XIAP but also inhibits cIAP1 and cIAP2 (cellular inhibitor of apoptosis proteins 1 and 2) by binding to the BIR3 domains of cIAP1, cIAP2, and XIAP. This dual inhibitory property gives it a unique advantage in anti-apoptotic mechanisms (24). Kadletz et al. verified that AZD5582 promoted apoptosis and inhibited tumor proliferation and migration in head and neck squamous cell carcinoma cells only by *in vitro* experiments (25). We demonstrated from both *in vivo* and *in vitro* experiments that AZD5582 inhibited the proliferation of hepatocellular carcinoma cells and significantly reduced tumor volume in mice.

In our study, we found that AZD5582 affects Ki-67 expression, which we believe may be related to the following aspects: 1) inhibition of XIAP activates the apoptotic pathway causing cells to enter into programmed death, thus reducing the overall Ki-67 expression level (26); 2) inhibition of XIAP leads to cell cycle arrest at the G1/S or G2/M checkpoints, which prevents the cells from efficiently entering into the proliferative phase, and further reducing Ki-67 expression (27); 3) XIAP affects cell proliferation by regulating signaling pathways, such as PI3K/Akt, MAPK/ERK, NF-κB, and its inhibition may lead to the inactivation of these pathways, thus affecting cell proliferation and Ki-67 expression (18). Hoefsmit et al. demonstrated that AZD5582 increased CD8+ T-cell proliferation and the secretion of cellular inflammatory factors (IFN-γ, TNF, IL-2, etc.) after presentation of tumor antigen by dendritic cells (28). This is similar to our findings. Our results showed that after the application of AZD5582, the expression level of CD8+ T cells was increased locally in the tumor, while the expression level of Foxp3+ regulatory T cells was decreased. However, a lack of resources is an obstacle for us in performing appropriate experiments to explore the underlying mechanism. A study demonstrated the drug toxicity of AZD5582 only by measuring whole blood indices in mice, without performing pathology on the vital organs of the mice (29). In our study, the low toxicity of AZD5582 was verified not only through hematology but also through pathology, which demonstrated its safety.

In conclusion, increased XIAP expression is associated with the progression of liver cancer after MWA treatment. The XIAP inhibitor AZD5582 mitigates the progression of liver cancer via inducing cell apoptosis and inhibiting cell proliferation *in vitro* and *in vivo*. The *in vivo* use of AZD5582 enhances infiltration of the CD8-positive effector T cells and shows preferable safety. This work might provide pre-clinical evidence for a novel adjunctive strategy for MMA treatment of HCC.

## Data Availability

The original contributions presented in the study are included in the article/supplementary material. Further inquiries can be directed to the corresponding authors.
